# Evaluation of the mental health status of community frontline medical workers after the normalized management of COVID-19 in Sichuan, China

**DOI:** 10.3389/fpsyt.2023.1198822

**Published:** 2023-08-10

**Authors:** Xiaoru Xu, Jianshu Zhang, Ronghua Fang, Hong Liu, Weihua Zhou, Yi She, Feng Liu, Xia Hong, Xuexue Deng

**Affiliations:** ^1^General Practice Ward, International Medical Center, General Practice Medical Center, West China Hospital, West China School of Nursing, Sichuan University, Chengdu, China; ^2^Yulin Community Health Service Center, Chengdu, China; ^3^Gaoxin Community Health Service Center, Chengdu, China; ^4^Nursing Department of West China Hospital, West China School of Nursing, Sichuan University, Chengdu, China

**Keywords:** Symptom Checklist-90 (SCL-90), normalized management, COVID-19, frontline medical workers, community, mental health

## Abstract

**Background:**

During the coronavirus disease 2019 (COVID-19) pandemic, community medical workers, as the primary enforcers of community control measures, undertook many tasks with high exposure risk, resulting in severe psychological pressure, anxiety, depression and other psychological problems. Gender, type of workers, education, marital status, working years and other demographic factors were affect the mental state of medical workers. Community frontline medical workers gradually returned to normal work and life after the normalized management of COVID-19, but heavy work and high psychological pressure may continue to affect them. Thus, our research team used the same psychological questionnaire to investigate the psychological status of community frontline medical workers after the normalized management of COVID-19 compared with the COVID-19 period.

**Methods:**

This was a cross-sectional study of community frontline medical workers in Sichuan, China, from February 6 to 17, 2023. Symptom Checklist-90 (SCL-90) and a self-designed questionnaire of demographic characteristics were provided to the participants point-to-point through a mobile network platform. Multiple logistic regression was used to analyze influencing factors related to community frontline medical workers’ psychology.

**Results:**

A total of 440 valid questionnaires were statistically analyzed, including 192 (43.64%) from doctors and 248 (56.36%) from nurses. There were 222 (50.45%) participants who were SCL-90 positive. The median total SCL-90 score of medical workers was 105.0 (IQR 95.00–123.75), which was higher than that during the COVID-19 period. The doctor’s median SCL-90 score was 108.5 (IQR 96.00–136.25), and the positive item score was 16.5; the nurse’s median score was 104.0 (IQR 94.00–119.50), and the positive item score was 12.0. Bachelor’s degree education, no fixed contract and working years (10–19 years, 20–29 years, 30–39 years) were independent influencing factors for community frontline medical workers’ psychology.

**Conclusion:**

After the normalized management of COVID-19, community frontline medical workers still suffered from psychological problems that were even more serious than those during COVID-19. Doctors were more likely to have psychological problems than nurses. In addition, the mental health status of community frontline medical workers was affected by education, type of contract and working years. Managers should pay attention to the mental health of these people.

## Background

Coronavirus disease 2019 (COVID-19) is the most rapidly spreading disease at present and exhibits rapid viral reproduction, widespread distribution and high prevalence (1, 2). COVID-19 poses a serious threat to public health in China and worldwide. Once infected, patients may experience generalized muscle and bone pain, cough, fever, severe pneumonia, hypoxia, and even death (3–6).

Community medical workers are primary health care providers, meeting the daily needs of the local population for prevention, rehabilitation, diagnosis and treatment of common and frequently occurring diseases, as well as health education (7). Community doctors and nurses are the first responders to community emergencies (8). During the COVID-19 pandemic, community medical workers, as the primary enforcers of community control measures, undertook many tasks with high exposure risk, such as searching and tracking the sources of infection, cutting off transmission routes, discovering and isolating close contacts, and screening key observation individuals (9). Approximately 4 million community medical workers are actively engaged in COVID-19 prevention and control in China (10).

In the early stage of COVID-19, many countries had to close their borders and implement domestic blockade management in hopes of curbing its spread. The Chinese government quickly adopted a variety of measures to control entry personnel, such as controlling entry, restricting public gatherings, wearing masks, washing hands frequently, and punishing those who endanger public health security. Through these measures, COVID-19 has been controlled effectively. To ensure the orderly social resumption of work and production, the government of China has adopted a more permissive approach to COVID-19, such as canceling nucleic acid certification, health codes and travel codes since December 8, 2022. To adjust the COVID-19 prevention and control plan from December 27, 2022, the focus of work has shifted from “preventing infection” to “protecting health and preventing serious diseases.” According to the current situation, “Class B and B tubes” have been implemented to actively optimize and improve control measures, with the aim of constantly making prevention and control work more scientific, accurate and effective; those infected with COVID-19 will no longer be quarantined, and close contacts will not be identified. Similarly, there are no high-risk or low-risk areas. With respect to entry persons and goods, quarantinable infectious disease control measures have been abolished, and the focus is strengthening services and safeguards (11).

The focus of COVID-19 prevention has changed, which does not mean the end of COVID-19, and the impact of COVID-19 on medical workers is not over. Studies have demonstrated that medical workers were prone to severe insomnia, anxiety, depression, and PTSD during COVID-19 (12, 13). Severe acute respiratory syndrome (SARS) took approximately 6 months from outbreak to end, but the psychological impact of the epidemic on people lasted much longer (14). A previous study found that some people still have symptoms of depression and anxiety at 30 months post-SARS (15, 16). Within 6 months following the COVID-19 outbreak, medical workers exhibited a higher prevalence of posttraumatic stress disorder (PTSD) than the general public (27% vs. 19%) (17). Therefore, negative emotions continue to affect medical workers (18).

Previous studies have found that frontline medical workers have invested significant time and effort during the COVID-19 period, resulting in severe psychological pressure, burnout, anxiety, pain, fear and depression (19–22). Our research team investigated the psychology of community frontline medical workers during the COVID-19 period, and the results showed that community frontline medical workers had similar psychological experiences (10). After the normalized management of COVID-19, although community frontline medical workers gradually returned to normal work and life, heavy work and high psychological pressure may continue to affect community frontline medical workers during the COVID-19 period, causing psychological problems. There are many studies on the psychological status researching of frontline medical workers, but few studies have examined the mental health of community frontline medical workers after the normalized management of COVID-19. To understand the psychological changes of community frontline medical workers after the normalized management of COVID-19 compared with the COVID-19 period. Our research team used the same psychological questionnaire to investigate the psychological status of frontline medical workers in the community to provide baseline data for the psychological intervention of community workers to managers, promoting targeted psychological intervention measures.

## Materials and methods

### Population and data

**FIGURE 1:**
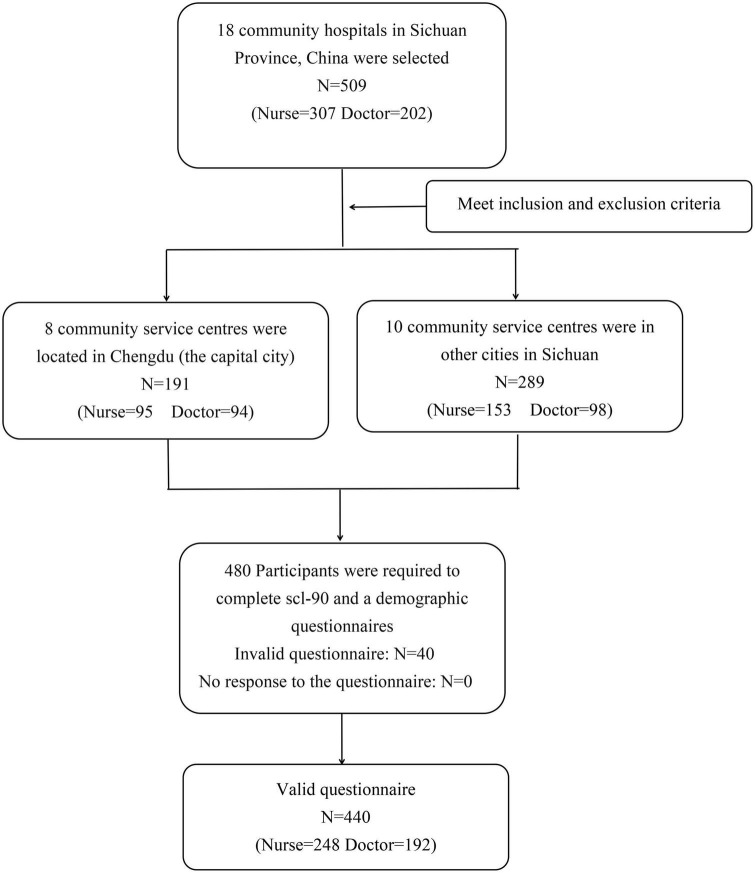
Flow chart of the data collection.

A cross-sectional study was used in this research. From 6 to 17 February 2023, the research team conducted a survey of all frontline medical workers at 18 community health service centers located in 11 cities in Sichuan Province who had participated in the previous survey from 8 to 18 February 2020 (10). In this study, community medical workers refer to community doctors and nurses, excluding other medical-related staff. There are 25–30 frontline medical workers per community health service center, for a total of 509 people. There were 480 people who met the inclusion and exclusion criteria, and all of them were included in the survey (Figure 1). The sample size of multivariate analysis is 10 times that of variables (23). In this study, 14 variables were analyzed, and the sample size was at least 140 cases. The Ethics Committee of the West China Hospital of Sichuan University approved this research. All participants signed informed consent forms. Survey data were anonymously coded to ensure that identifying information remained confidential.

The inclusion criteria were as follows: (1) long-term labor signing with the community; (2) ≥1 year of frontline work experience in the community; (3) obtaining the professional qualification certificate of doctors or nurses; and (4) agreement to participate in this survey. The exclusion criteria were as follows: participants who were taking antipsychotic medications, such as anti-anxiety medications and antidepressants.

For the purpose of our investigation, we gave participants two questionnaires: the Symptom Checklist 90 (SCL-90) was the same as that of Zhang et al. (10) and a self-designed questionnaire of demographic characteristics that had fewer items “whether have participated in major emergencies” than the questionnaire of Zhang et al. (10). Data were collected through point-to-point online surveys using electronic questionnaires and were set up for one person to fill in once. Before handing out the questionnaire, we asked the respondents online in a unified language to determine whether they met the inclusion and exclusion criteria and then handed out questionnaires point-to-point. To complete the e-questionnaire, participants needed to read the electronic invitation letter and click the option “I agree to participate in the survey” to continue to access the questionnaire. Mandatory items were set in the questionnaire, which cannot be submitted until completed, to ensure the integrity of data collection. Before the collected data were included in the database for analysis, invalid questionnaires were eliminated to ensure objective and true analysis data. Invalid questionnaires included questionnaires that took less than 8 min to complete (in the preliminary experiment, it took at least 8 min to complete the questionnaire) and repeated questions that gave inconsistent answers (we set the same questions in the questionnaire and switched the option positions). The database was built using EpiData 3.1 software (EpiData–Comprehensive Data Management and Basic Statistical Analysis System, EpiData Association, Odense, Denmark) and then double-checked. Invalid questionnaires were excluded from the included data.

### Outcomes

Psychological states were assessed using the SCL-90 scale. SCL-90 is a psychosomatic screening scale developed by Derogatis (24). The SCL-90 scale currently used in China was translated by Wang (25). The Cronbach’s alpha coefficient of the SCL-90 ranges from 0.77 to 0.99 (24) and is used to distinguish patients with psychosomatic diseases from patients without psychosomatic diseases (26). It reflects the mental health status of the individual in the most recent week (25). The SCL-90 includes 90 items that are negatively entered, reflecting 10 diagnoses: somatization (SOM, 12 items), obsessive-compulsive disorder (O-C, 10 items), interpersonal sensitivity (I-S, 9 items), depression (DEP, 13 items), anxiety (ANX, 10 items), anger-hostility (HOS, 6 items), phobic anxiety (PHOB, 7 items), paranoid ideation (PAR, 6 items), psychoticism (PSY, 10 items), and others (reflecting sleep and diet, 7 items). The severity of each item is rated on a 5-point Likert scale: 1 = not at all, 2 = a little bit, 3 = moderate, 4 = quite a bit, and 5 = most severe. A valid SCL-90 questionnaire means that 90 items have been completed. The total score (T-S) is the sum of 90 items, the score of each factor refers to the total score of the questions contained on the factor subscale, and a factor score ≥2 is considered positive. The number of factor-positive items refers to the total number of items scored ≥2, and the number of negative items is the total number of all items with a score = 1. In the investigation population, if the total score was ≥160, the number of positive items was ≥43, and if the average score for any of these factors was ≥2, it was defined as SCL-90 positive, indicating psychological health problems (25).

### Covariates

We determine items that might have a psychological impact as demographic data by reviewing the literature. The socioeconomic and demographic factors selected were gender, age, type of workers, professional title, work position, have children, education, marital status, history of chronic disease, working years, type of contract, personal monthly income, family monthly income and previous occupational risk of exposure to COVID-19. Professional title refers to medical workers who have worked for a certain number of years and have passed the national unified examination to obtain the certificates recognized by the state (including junior, intermediate, and subsenior above), reflecting their technical level and working ability. Type of contract is the form of Chinese workers and the unit sign a contract, divided into fixed-term contract and no fixed-term contract. In the same unit, a work time of 9 years or less signed a fixed term contract. The fixed term contract specifies the termination time of the contract, and there is no other legal relationship between the unit and Chinese workers after the expiration of the contract. Working more than 9 years is signed with no fixed term contract. No fixed-term contract means that both parties to the contract have not agreed on the termination time; only in the event of legal termination, such as retirement or death, will labor relations be terminated. Occupational exposure depends on workplace and the direct or indirect distance of contact with the patient. For example, the occupational exposure risk of working in fever consultation rooms or fever consultation sites in epidemic areas was higher than that of home isolation and home follow-up of close contacts (9).

### Analyses

SPSS software (Version 26.0. IBM Inc., Armonk, NY) was used for statistical analyses. Quantitative data (score data of each dimension of SCL-90) were described by median and interquartile spacing (IQR), and two independent sample tests (Mann-Whitney U test) in non-parametric tests were used to analyze the differences in SCL-90 scores on various dimensions between doctors and nurses and between this study and related studies during the COVID-19 period. Qualitative data are presented as frequencies and percentages, and the ×2-test was used to analyze the positive difference in SCL-90 among different demographic data. Logistic regression was used to analyze the factors associated with the psychological status of frontline medical workers. Whether it is positive for SCL-90 as the dependent variable, and all variables of demography are the independent variable. The odds ratios and 95% confidence intervals (CIs) were calculated by logistic regression. A *p*-value < 0.05 was considered significant.

## Results

### Demographic characteristics of the participants and SCL-90 scores

A total of 480 community frontline medical workers participated in the investigation in Chengdu, Sichuan. The number of complete questionnaires was 440, and the effective completion rate of the questionnaire was 91.67%. The majority of participants were female and between 30 and 39 years old, married (85.91%), had junior professional titles (44.9%), had children (79.09%), had obtained a bachelor’s degree (60.91%), and had a high previous occupational exposure risk (35.91%). The median total SCL-90 score of community frontline medical workers was 105.0 (IQR 95.00–123.75). The number of SCL-90-positive community frontline medical workers was 222 (50.45%), and the number of positive items was 13.0 (IQR 4.00–28.00). There was statistical significance in the association of SCL-90-positive medical workers with different types of workers, marital status, working years and family monthly income (*p* < 0.05). Doctors who were divorced, had 30–39 working years and had a family monthly income < 3000 yuan (1 yuan≈0.1400 USD) were more likely to be SCL-90 positive (Table 1).

**TABLE 1:** Demographic characteristics of the community frontline medical workers and SCL-90.

Variables	*n*(%)	*n*(%)*	χ *^2^*	*P*
Gender			0.148	0.094
Male	48(10.91)	26(54.17)		
Female	392 (89.09)	196(50.0)		
Age,y			3.633	0.290
<30	108 (24.54)	44(40.74)		
30–39	238 (54.09)	122(51.26)		
≥40	94 (21.36)	56(59.57)		
Type of workers			2.184	0.002
Doctor	192 (43.64)	112(58.33)		
Nurse	248 (56.36)	110(44.35)		
Professional title			4.841	0.221
None	24 (5.45)	8(33.33)		
Junior	194 (44.09)	88(45.36)		
Intermediate	182 (41.36)	100(54.95)		
Subsenior or above	40 (9.09)	26(65.00)		
Work position			1.996	0.893
None	368 (83.64)	178(48.37)		
Medical/Nursing team leader	30 (6.82)	18(60.0)		
Head doctor/Nurse	22 (5.00)	14(63.64)		
Others	20 (4.55)	12(60.00)		
Have children			0.537	0.464
Yes	348 (79.09)	180(51.72)		
No	92 (20.91)	42(45.65)		
Education			6.084	0.245
Junior college	172 (39.09)	70(40.70)		
Bachelor’s degree	268 (60.91)	152(56.72)		
Marital status			0.506	0.000
Unmarried	56 (12.73)	26(46.43)		
Married	378 (85.91)	192(50.79)		
Divorced	6 (1.36)	4(66.67)		
History of chronic disease			1.693	0.136
Yes	30 (6.82)	20(66.67)		
No	410 (93.18)	202(49.27)		
Working years, y			4.224	0.032
1–9	140 (31.82)	64(45.71)		
10–19	220 (50.00)	114(51.82)		
20–29	66 (15.00)	32(48.48)		
30–39	14 (3.18)	12(85.71)		
Type of contract			6.290	0.100
Fixed term contract	394 (89.55)	188(47.72)		
No fixed contract	46 (10.45)	34(73.91)		
Personal monthly income, yuan^△^			1.577	0.753
<3000	16 (3.63)	8(50.00)		
3000–4999	164 (37.27)	78(47.56)		
5000–7999	224 (50.91)	118(52.68)		
8000–9999	34 (7.73)	16(47.06)		
≥10000	2 (0.50)	2(100.00)		
Family monthly income, yuan^△^			4.379	0.029
<3000	20 (4.55)	16(80.00)		
3000–4999	128 (29.09)	66(51.56)		
5000–7999	138 (31.36)	62(44.93)		
8000–9999	82 (18.64)	42(51.22)		
≥10000	72 (16.36)	36(50.00)		
Occupational exposure			2.184	0.507
Moderate	152 (34.55)	84(55.26)		
High	130 (29.55)	56(43.08)		
Very high	158 (35.91)	82(51.90)		

*The number (percentage) of people who were SCL-90 positive. △Yuan is a unit of measure for Chinese yuan (RMB), 1 yuan≈0.1400.

### Comparison of SCL-90 factor scores between doctors and nurses

The doctor’s median SCL-90 score was 108.5 (IQR 96.00–136.25), and the positive item score was 16.5 (IQR 4.25–33.00); the nurse’s median score was 104.0 (IQR 94.00–119.50), and the positive item score was 12.0 (IQR 3.00–22.00). There were significant differences between doctors and nurses in total median score, I-S, DEP, ANX, PAR, and PSY (*p* < 0.05). Doctors had higher scores than nurses (Table 2).

**TABLE 2:** Comparison of SCL-90 factor scores between doctors and nurses (IQR).

Factors	Doctors (*n* = 192) median (IQR)	Nurses (*n* = 248) median (IQR)	*Z*	*P*
Total	108.5(96.00–136.25)	104(94.00–119.50)	−2.063	0.039
SOM	1.17(1.00–1.33)	1.08(1.00–1.33)	−0.778	0.437
O-C	1.30(1.10–1.90)	1,30(1.10–1.60)	−1.455	0.146
I-S	1.20(1.00–1.58)	1.10(1.00–1.38)	−2.265	0.024
DEP	1.17(1.00–1.56)	1.08(1.00–1.25)	−2.501	0.012
ANX	1.20(1.00–1.50)	1.10(1.00–1.30)	−2.207	0.027
HOS	1.17(1.00–1.50)	1.17(1.00–1.33)	−0.857	0.392
PHOB	1.14(1.00–1.43)	1.07(1.00–1.29)	−1.397	0.162
PAR	1.00(1.00–1.33)	1.00(1.00–1.17)	−2.614	0.009
PSY	1.09(1.00–1.27)	1.00(1.00–1.16)	−2.291	0.022
Others	1.29(1.00–1.57)	1.14(1.00–1.43)	−1.228	0.219

SOM, somatization; O-C, obsessive compulsiveness; I-S, interpersonal sensitivity; DEP, depression; ANX, anxiety; HOS, anger hostility; PHOB, phobic anxiety; PAR, paranoid ideation; PSY, psychoticism; Others, reflecting sleep and diet.

### Comparison of SCL-90 factor scores between community frontline medical workers after the normalized management of COVID-19 and during the COVID-19 period

In the comparison of the median SCL-90 scores between community frontline medical workers during COVID-19 (11) and after the normalized management of COVID-19, the total score (1.28, 1.18 *p* < 0.05), O-C (1.45, 1.30 *p* < 0.05), I-S (1.29, 1.11 *p* < 0.05), ANX (1.25, 1.10 *p* < 0.05), PHOB (1.24, 1.00 *p* < 0.05) and PAR (1.17, 1.00 *p* < 0.05). These factors were higher than during the COVID-19 period, and the difference was statistically significant (*p* < 0.05) (Table 3).

**TABLE 3:** Comparison of SCL-90 factor scores between community frontline medical workers after the normalized management of COVID-19 and during the COVID-19 period.

Factors	This study (*n* = 440, median)	Zhang′s study (*n* = 450, median)	*Z*	*P*
Total	1.28	1.18	2.382	0.017
SOM	1.23	1.17	−0.795	0.427
O-C	1.45	1.30	2.851	0.004
I-S	1.29	1.11	3.720	0.000
DEP	1.27	1.15	1.853	0.064
ANX	1.25	1.10	5.878	0.000
HOS	1.38	1.17	1.025	0.305
PHOB	1.24	1.00	9.354	0.000
PAR	1.17	1.00	8.099	0.000
PSY	1.15	1.05	0.405	0.685
Others	1.29	1.29	−0.997	0.319

SOM, somatization; O-C, obsessive compulsiveness; I-S, interpersonal sensitivity; DEP, depression; ANX, anxiety; HOS, anger hostility; PHOB, phobic anxiety; PAR, paranoid ideation; PSY, psychoticism; others, reflecting sleep and diet.

### Comparison of the positive items of each factor between doctors and nurses

Comparing the top three positive items of the SCL-90 factor of community doctors and nurses, the top three positive items among nurses were O-C, I-S and others, and the top three positive items among doctors were O-C, I-S and DEP (Table 4).

**TABLE 4:** Top three positive items among different doctors and nurses.

Type of worker	First ranked factor *n*(%)	Second ranked factor *n*(%)	Third ranked factor *n*(%)
Doctors	O-C 41(73.21)	I-S 30(53.57)	DEP 29(51.79)
Nurses	O-C 38(69.09)	I-S 26(47.27)	Others 24(43.64)

O-C, obsessive compulsiveness; I-S, interpersonal sensitivity; DEP, depression; Others, reflecting sleep and diet.

### Logistic regression analysis of multiple factors related to the psychological status of community frontline medical workers

Multiple logistic regression was used to analyze factors influencing the psychological status of community frontline medical workers. Whether it is positive for SCL-90 (whether there is a psychological health problem) as the dependent variable, and the purpose of this study was to explore the psychological influencing factors of medical workers, so all variables of demography were included in the model for analysis. Bachelor’s degree education, no fixed contract and working years (10–19 years, 20–29 years, 30–39 years) were identified as independent risk factors for positive symptoms on the SCL-90 in community frontline medical workers (Table 5).

**TABLE 5:** Analysis of positive influencing factors of the SCL-90 for community frontline medical workers.

Variable		*b*	Sb	Wald	*P*	OR (95%CI)
Education	Junior college = 1					
	Bachelor’s degree = 2	0.698	0.203	11.807	0.001	2.009(1.350, 2.991)
Type of contract	Fixed term contract = 1					
	No fixed contract = 2	0.700	0.291	5.773	0.016	2.014(1.138, 3.564)
Working years, y	1–9 = 1					
	10–19 = 2	2.136	0.801	7.108	0.008	0.118(0.025, 0.568)
	20–29 = 3	1.987	0.797	6.221	0.013	0.137(0.029, 0.653)
	30–39 = 4	2.334	0.838	7.752	0.005	0.097(0.019, 0.501)
Constant		0.510	0.981	0.270	0.603	

## Discussion

It has been 3 years since the outbreak of COVID-19, which is characterized by widespread transmission and high infectivity. The main route of transmission is through respiratory droplets and contact, and the population is generally susceptible (27). Although the fatality rate of COVID-19 is lower than that of SARS and Middle East Respiratory Syndrome (MERS), its impact on medical workers is not diminished (28). Previous research found that medical workers who experienced SARS and MERS were prone to psychological problems that did not disappear with time (14, 29). We found that doctors and nurses (58.33% vs. 44.35%) were persistently SCL-90 positive, and this positivity rate is higher than that previously reported by Zhang (34.8% vs. 20.4%) (10). During the COVID-19 period, doctors, compared with nurses, assumed more positions with higher exposure risk in the community, which also gave doctors to witness the effect of the virus more closely, making them more prone to negative emotions and PTSD. In addition, frequent contact with infected patients and worrying about being infected can increase the risk of PTSD (30). Therefore, doctors seem to suffer more psychological shocks than nurses. We found that divorced community frontline medical workers were more likely to be SCL-90 positive, which is likely related to family support. Walton (31) found that subjective emotional comfort and objective material support brought by family support can relieve psychological stress; conversely, a lack of communication with partners and the absence of children can increase psychological stress. Medical workers with ≥30 years have more work experience, so they undertook heavier work responsibilities after the normalized management of COVID-19 in the community, and they may have suffered from greater work pressure and less sleep. Long-term sleep deprivation can cause both physical fatigue and may also be considered an indicator of physical and mental health (32). Income has a positive effect on individual mental health (33), and higher income gives health workers better psychological defenses (34). Therefore, the psychological condition of community frontline medical workers deserves continuous attention. Managers should reasonably adjust working hours or community frontline medical worker responsibilities; in addition, managers could also stimulate the intrinsic motivation of all medical workers by means of compensation and reward.

In addition, our investigation found that after the normalized management of COVID-19, the scores of various factors and the incidence of psychological disorders among doctors were higher than those among nurses, which is inconsistent with Wu (35). Community doctors, as the backbone of the primary medical and health team (36), will return to normal work and life after COVID-19 has been normalized. Although they will not face high-intensity prevention and control work after the normalized management of COVID-19, COVID-19 may still break out on a small scale, and community doctors bear more responsibilities than nurses in the possible small scale of COVID-19 (37). These reasons may lead to doctors being more prone to anxiety, depression, interpersonal sensitivity, paranoia, psychosis and other psychological problems. In contrast, nurses’ social status improved after COVID-19 (38). As an important resource for individuals to cope with stress, social support can play a positive role in the stress response (39). Therefore, more attention should be given to doctors; in particular, for doctors who have been involved in high-risk jobs with occupational exposure, proper rest and psychological counseling can promote recovery from work effectively.

We found that the median score for each of the SCL-90 factors was higher than that of Zhang (10). After the normalized management of COVID-19, there was “zero growth” of COVID-19 in China, and community frontline medical workers gradually resumed their normal work and life. However, “zero growth” does not mean “zero risk” (40). Community frontline medical workers still have to deal with the possible outbreak of COVID-19, and their spirits are still in a state of tension and are unable to relax completely. In addition, due to multiple contacts with contaminated patients in the early stage, the viral load is high, the environmental pressure is great, the function of the immune system is affected (41, 42), the adaptability of the body is decreased, and the body is worried about its own health, resulting in increased psychological pressure. These may be the reasons why community frontline medical workers have more psychological problems than during the COVID-19 period.

After the normalized management of COVID-19, the top two SCL-90 scores for doctors and nurses were the same, including O-C and I-S. The difference is that the third highest score for doctors was DEP, whereas the third highest score for nurses was Others. Community institutions are the first line of defense for residents’ health, whether during the COVID-19 period or after the normalized management, and community frontline medical workers must undertake significant work after normalization (8). O-C symptoms included compulsive washing, examination and hoarding. With the changed policy, people with COVID-19 will not be quarantined, which will increase the risk of infection for community frontline medical workers and cause concern about family members being infected. Fear of infection (FoBI) is a normal psychological reaction that is increasingly recognized (43). The psychology of FoBI may lead to compulsive behaviors such as repeated hand washing to avoid infection, which may account for the high O-C among frontline health care workers in the community. With the comprehensive resumption of work and school, there should be a shift from “all that is receivable” and “all that should be treated” to “all that should be checked” and “all that is willing to be checked” for key groups in the region. In some areas, the incorrect interpretation of the policy increased complaints of the general public, manifested in the vent of discontent to community frontline medical workers, placing workers in a difficult position. Because the COVID-19 prevention policy was not understood and accepted by the public, tension was created between frontline medical workers and the public. DEP was characterized by a lack of enjoyment in life and work, low energy, worthlessness, helplessness, hopelessness and self-abandonment. Thus, relevant departments should correctly implement prevention and control policies after the normalized management of COVID-19 and publicize mass prevention and control so that the community is fully aware of the importance and necessity of prevention and control work. Media such as WeChat, radio and leaflets could be used to broadcast information to reduce the strained relationships faced by community frontline medical workers. Doctors may have more self-fulfilling psychological needs than nurses (44). They hope to wait for more development and recognition after the normalized management of COVID-19, which may lead to more prominent depression among doctors, while nurses are more likely to be affected by work and interpersonal relationships, manifested in sleep and diet problems.

Education, type of contract and working years were independent risk factors for SCL-90 positivity in community frontline medical workers. Regarding the level of education, the risk of SCL-90-positive symptoms in medical workers with a bachelor’s degree was higher than that in medical workers with a college degree (OR = 2.009; 95% CI = 1.350–2.991). Medical workers with higher education often have a complete knowledge system and learning ability, and this group tends to undertake more community work. After the normalized management of COVID-19, in addition to their daily work, they also shoulder more additional work, such as community publicity and psychological counseling, and their workload is not reduced. Long-term heavy workloads have different degrees of impact on medical workers (45). In addition, there is not enough compensation to reflect pay, resulting in this group of frontline medica workers being more prone to psychological problems (46). Frontline medical workers in the community with no fixed term contract were also at higher risk of positive symptoms than those with fixed term contract (OR = 2.014; 95% CI = 1.138–3,564). Medical workers with no fixed contract usually have longer service periods in the community; they have accumulated extensive work experience and may assume positions (47), shoulder more responsibilities and pressure, and work effort and return may not meet expectations, leading to more psychological problems (48, 49). Community frontline medical workers with 20–29 years of service were more likely to suffer psychological problems (OR = 0.137; 95% CI = 0.029–0.653). This result is similar to previous studies (50, 51), which may be related to this group being facing the position of supporting the elderly and taking care of children at the same time. Therefore, community managers should pay attention to the mental health of frontline medical workers, especially frontline medical workers with high education, no fixed contract and more than 10 years of work, giving care, reasonably arranging work, and promoting their mental health.

Community frontline medical workers still had prominent psychological problems after the normalized management of COVID-19 was more serious than during COVID-19. Doctors were more likely to have psychological problems than nurses. In addition, the mental health status of community frontline medical workers was affected by education, type of contract and working years. Managers should pay more attention to the mental health of these people and take measures to improve their psychological problems.

### Limitations

In this study, we used the same psychological scale to investigate the same area and community health service center after the normalized management of COVID-19. Through continuous investigation comparing the psychological status of medical workers during the COVID-19 period and after normalized management, the research results were more representative. This study had several limitations. First, the participants in this study were not all the same as those in the previous study. Second, this was a cross-sectional analysis with inherent design limitations; the psychology of surveyors will fluctuate in different periods (52), and the survey results will also change with time. Finally, this study was conducted in only one province, Sichuan, and the subjects were only doctors and nurses, so the data representativeness was limited. Based on the findings of this study, our team will continue to pay attention to the psychological state of community frontline medical workers in the future and may conduct relevant studies after the normalized management of COVID-19 for 1 year.

## Conclusion

This study found that community frontline medical workers continued to suffer psychological problems after the normalized management of COVID-19, even more serious than during the COVID-19 period. Doctors were more likely to have psychological problems than nurses. In addition, the mental health status of community frontline medical workers was affected by education, type of contract and working years. Managers should pay more attention to the mental health of these frontline medical workers and take effective actions to solve the psychological problems for different groups.

## Data availability statement

The raw data supporting the conclusions of this article will be made available by the authors, without undue reservation.

## Author contributions

XD conceptualized the project, undertook the project administration and supervision, and analyzed and interpreted the data. XX, JZ, HL, WZ, YS, XH, and FL performed the investigation. XX prepared the original draft. XD and RF reviewed and edited the manuscript. All authors read and approved the final manuscript.
